# International medical graduates amongst pathology leadership roles: more than meets the eye

**DOI:** 10.1016/j.acpath.2025.100207

**Published:** 2025-07-28

**Authors:** Stephen M. Smith, Vinita Parkash

**Affiliations:** Department of Laboratory Medicine & Pathobiology, The University of Toronto, Toronto, Ontario, Canada; Anatomic Pathology, Toronto General Hospital, University Health Network, Toronto, Ontario, Canada; Department of Pathology, Yale University School of Medicine, New Haven, CT, United States; Yale University School of Public Health, New Haven, CT, United States

**Keywords:** international medical graduates, leadership

We commend Drs. Azemi and Parra-Herran for their recent publication^1^ on international medical graduate (IMG) representation in the academic workforce, departmental and society leadership. They rightly identify the underrepresentation of international medical graduates (IMGs) in leadership roles in pathology. This is in line with our previous argument.^2^

However, we feel that the inference of representative leadership at the departmental level versus nonrepresentative leadership at the professional society level is perhaps overstated. Although the authors reflect on the differences in timeframes between data collection pertaining to Department Chairs and Society Leadership as a potential source of bias, we posit that this difference between the two cohorts makes any comparison impossible. We suspect that if 30-year trends for Chairs were investigated, or if person-years between IMGs and United States and Canadian medical graduates (USCMGs) were used as the metric for comparison, the inferences of this study may well be reversed, especially considering the marked difference in terms between department Chairs and Presidents. Certainly, if one were to only look for the year 2024, the numbers would be 7:20 (department chairs) vs 1:4 (society presidents), a likely nonsignificant difference. Major professional societies (USCAP, CAP, ASCP) have made concerted efforts for diverse representation in leadership; three of the last 10 USCAP Presidents over the last decade have been IMGs. The CAP will welcome its first IMG president this year (2025)–Dr. Quihui “Jim” Zhai.^3^

Other factors that limit comparison between two cohorts include (1) the inherent structural differences between organizations; and (2) the processes for selection of organizational leadership. The Association for Academic Pathology (AAPath) is a society of departments, represented by individuals in select leadership roles; other societies are composed of individual pathology members. Some societies select presidents by popular vote of membership (e.g. the CAP); others do not. Some universities require the exclusion of pathology representation from Chair selection committees, to avoid potential bias in favor of the “internal candidate,” others do not. The motivations between selecting leadership in these two settings typically differ.

In our view, the generalizability of the inferences from these data to the specialty of pathology is also perhaps overstated. Twenty academic institutions are arguably too small a sample to represent the population of academic pathology departments; 4 “generalist societies” unlikely representative of the societies in pathology, the majority of which are now subspecialty-focused (e.g. ISGyP, ISUP, GUPS, GIPS, DAPA etc.). It is unclear as to why or how these institutions or societies were chosen for study. We note that the data obtained are highly dependent on the accuracy of the publicly available data obtained via the internet, which may not be up to date. The geographic distribution of the institutions selected for study is somewhat broad, but there may be regional differences in the data which limits generalizability.

We also wish to highlight some other interesting elements in the authors’ (supplementary) data regarding IMG representation in the academic workforce, which perhaps inform the differences in representation at the leadership level. There is marked variability in the percentage of IMG faculty by institution, ranging from a low of 17% (MGH) to 67% (MD Anderson). 35% (7/20) of academic departments show less than 25% IMG faculty, while 40% (8/20) show more than 40% IMG faculty, with four institutions showing IMG representation of over 50%. Furthermore, if molecular and hematopathology, arguably subspecialties within anatomic pathology, are removed from the clinical pathology groups, the data displays a curious disparity of IMG representation between clinical pathology (6–21% IMGs) relative to anatomic (26–50% IMGs) pathology. These numbers raise concerns about institutional “medical inferiority bias” against IMGs in some institutions, and perhaps in some medical specialties. We have identified a segregation effect of IMGs into specialties, subspecialties and different-tier medical schools as an outcome of bias against IMGs.^2^^,^^4^ Career path-dependence disparity amongst IMGs, including ascension through leadership or career trajectory, is rooted in early inequity and class assignment.

Finally, we note an interesting finding: female IMGs are dramatically unrepresented amongst pathology chairs and society leadership. We know of several anecdotal instances; however, a formal study investigating the evolution and development of pathology leadership would further qualify this point. It would be particularly interesting to study how intersectionality is present in the hierarchical structures of pathology departments and societies, and the reasons underpinning this phenomenon.
